# Kai-Xin-San Protects Depression Mice Against CORT-Induced Neuronal Injury by Inhibiting Microglia Activation and Oxidative Stress

**DOI:** 10.1155/2022/5845800

**Published:** 2022-10-19

**Authors:** Guiqin Bai, Shangwen Jing, Huimin Cao, Yiqi Qiao, Gongchan Chen, Lining Duan, Yuna Yang, Min Li, Weirong Li, Xiang Chang, Cong Yang, Qi Wang

**Affiliations:** ^1^Science and Technology Innovation Center, Guangzhou University of Chinese Medicine, Guangzhou, China; ^2^Basic Medical Sciences & Forensic Medicine of North Sichuan Medical College, Nanchong, Sichuan Province, China; ^3^Clinical Medical College of Acupuncture Moxibustion and Rehabilitation, Guangzhou University of Chinese Medicine, Guangzhou, China; ^4^Xi'an Hospital of Traditional Chinese Medicine, Xi'an, Shaanxi Province, China

## Abstract

**Objective:**

Traditional Chinese medicine formula Kai-Xin-San (KXS) is used to treat psychiatric disorders, especially in anxiety and depression. However, the precise molecular mechanism of action remains unclear. In this study, we investigated the antidepressant effect of KXS on inhibiting inflammation and oxidative stress in corticosterone (CORT)-induced depression.

**Methods:**

The therapeutic efficacy of KXS was evaluated in a mouse model of depression induced by CORT. Behavioral tests were conducted to evaluate the effectiveness of KXS in treating depressive-like behavior. Nissl staining and *β*-galactosidase staining were used to assess the effects of KXS on neuronal injury in depressed mice. To screen key potential therapeutic targets of KXS, transcriptome sequences and data analysis were performed. Then, Iba1 immunofluorescence staining and their relative inflammatory factors mRNA expression were conducted to assess the effect of KXS in inhibiting microglial inflammation activation response. Concurrently, the measurement of 4-Hydroxynonenal (4-HNE) immunohistochemistry staining, malondialdehyde (MDA), superoxide dismutase (SOD), and reactive oxygen species (ROS) were performed to evaluate the effect of KXS on anti-oxidative stress of depression *in vivo*. Besides, nitric oxide (NO), relative inflammatory factors mRNA expression, JC-1 staining, and ROS were used to evaluate the effect of KXS by lipopolysaccharide (LPS)/interferon-gamma (IFN*γ*)-induced BV2 cells.

**Results:**

KXS significantly relieved the depressive-like symptoms induced by CORT, as well as ameliorating the neuronal damage, which decreased microglia inflammatory activation response of IL-1*β*, IL-6, and tumor necrosis factor *α* (TNF*α*) *in vivo or in vitro* too. Transcriptome Sequencing and Data Analysis showed that KXS mainly by regulating immune system and transduction pathways decreased CORT-induced depression in mice. And showed that there were 19 Principal components and 10 genes in the main regulatory position with the strongest correlation in depression mice. Meanwhile, KXS effectively decreased senescence, the expression of 4-HNE, MDA content, and the production of ROS, while increasing the SOD activity in CORT-induced mice. Besides, KXS significantly reversed the mitochondrial membrane potential loss and excessive ROS production in LPS/IFN*γ*-induced BV2 cells.

**Conclusion:**

Our research suggested that KXS might protect depressed mice against CORT-induced neuronal injury by inhibiting microglia activation and oxidative stress.

## 1. Introduction

Depression is a psychiatric illness that creates a persistent depressed mood, negative thoughts, and fatigue [1]. It is currently the leading cause of disability worldwide, with over 300 million people affected by the disease [2]. The current treatment of depression is mainly based on drug therapy. However, these single-target antidepressants are characterized by the drawbacks of lagging, side effects, poor compliance, and inefficient treatment [3]. Therefore, it is urgent to develop drugs that have therapeutic effects on multiple pathogenesis [4, 5].

Traditional Chinese medicine (TCM) has special advantage in the therapy of emotional symptoms with complex symptoms because of its wide range of chemical components, as well as multiple targets and pharmacological effects [6]. Kai-Xin-San (KXS), derived from Qian-Jin-Yao-Fang written by Sun Si-Miao during the Tang Dynasty, is a famous TCM formula that has been widely applied for depression treatment [7, 8]. In recent times, many researches have displayed that KXS has potential antidepressant effects. KXS administration enhanced synaptogenesis by enhancing synaptic protein expression [9], and elevated the contents of norepinephrine (NE), 5-hydroxytryptamine (5-HT), and dopamine (DA) in mice significantly [10]. As a result, this could explain its antidepressant effect. KXS may also exert antidepressant effects by regulating signaling pathways for lipid metabolism disorders [11]. However, the exact antidepressant mechanism that underlies the effect of KXS remains unclear.

The hypothalamic-pituitary-adrenal (HPA) axis dysfunction, inflammation and oxidative stress are the three major pathophysiological hypotheses in the development of depression [11]. An important pathophysiological feature of depression is the dysfunction of the HPA axis [12]. Hypercortisolemia and dysregulation of the HPA axis are often found in patients with depression [13]. The administration of corticosterone (CORT) increases microglia number, while blocking CORT production or action abrogates acute or chronic stress-induced microglial proliferation and activation [14]. There is growing evidence that activated microglia are chronic sources of multiple neurotoxic factors, including tumor necrosis factor *α* (TNF*α*), nitric oxide (NO), interleukin-1*β* (IL-1*β*), reactive oxygen species (ROS), and driving progressive neuronal injury [15]. Unfortunately, the brain is one of the most vulnerable organs to ROS damage, which may account for ROS involvement in depression. Inflammation could initiate oxidative stress [16]. *Vice versa*, oxidative stress also plays a critical role in the development of inflammation [17]. These results showed a link between the HPA axis, inflammation, and oxidative stress, and the interactions had a crucial impact on depression. Therefore, anti-inflammation and oxidative stress were supposed as the crucial therapeutic stratagems for depression. Nevertheless, the research on the effects of KXS in anti-inflammation, especially anti-oxidative stress remains largely unexplored in depression.

In this study, we attempted to investigate the underlying mechanisms for treating depression with KXS. First, we evaluated the therapeutic efficacy of KXS in depression using the CORT-induced depression mouse model. Secondly, we examined the effect of KXS on inhibiting microglia activation and proinflammatory response by Iba1 immunofluorescence staining and relative inflammatory factor mRNA expression. Finally, we performed the measurement of *β*-galactosidase staining, 4-HNE immunohistochemistry staining, JC-1 staining, malondialdehyde (MDA), superoxide dismutase (SOD), NO assay and ROS to assess the effect of KXS on anti-oxidative stress. Our research provided new evidence on the effect and underlying mechanism of KXS on depression treatment.

## 2. Materials and Methods

### 2.1. Drugs Preparation

The KXS contains four herbs: Ginseng, hoelen, polygala and acorus (Table 1), which were mixed in the ratio of 3 : 3 : 2 : 2 [17–19]. They were provided by the First Affiliated Hospital Pharmacy Room of Guangzhou University of Traditional Chinese Medicine (Guangzhou, China) and identified by the pharmacist Dr. He Zhu. Ginseng (90 g), hoelen (90 g), polygala (60 g), and acorus (60 g) were cut into small pieces before being mixed in together. KXS extraction steps and quality control were performed following our previous study [17–19]. Finally, KXS was condensed into an active drug with a concentration of 0.56 g/ml and obtained 420 *μ*l of volatile oil from acorus. Then, the acorus volatile oil combined with the concentrated liquid. All extracted drugs were stored in the refrigerator at −20°C.

### 2.2. Animal Groups

All the animals were purchased from Guangdong Medical Laboratory Animal Center (Guangzhou, China) and authorized by the Guangzhou University of Chinese Medicine Animal Ethics Committee (approval number: 20210112003). A total of 60 male Swiss mice (23–26 g) were performed in accordance with the principles and guidelines of the National Institutes of Health Guide for the Care and the Use of Laboratory Animals. They were fed and watered regularly at Specific Pathogen-Free (SPF) raising conditions. The mice were allocated into five groups using complete randomization by the random number table (*n* = 12/group): Control group (CON), CORT model group (CORT), KXS low dosage group (KXSL), KXS high dosage group (KXSH), and Fluoxetine group (FLX).

### 2.3. Treatment

**Figure 1:**
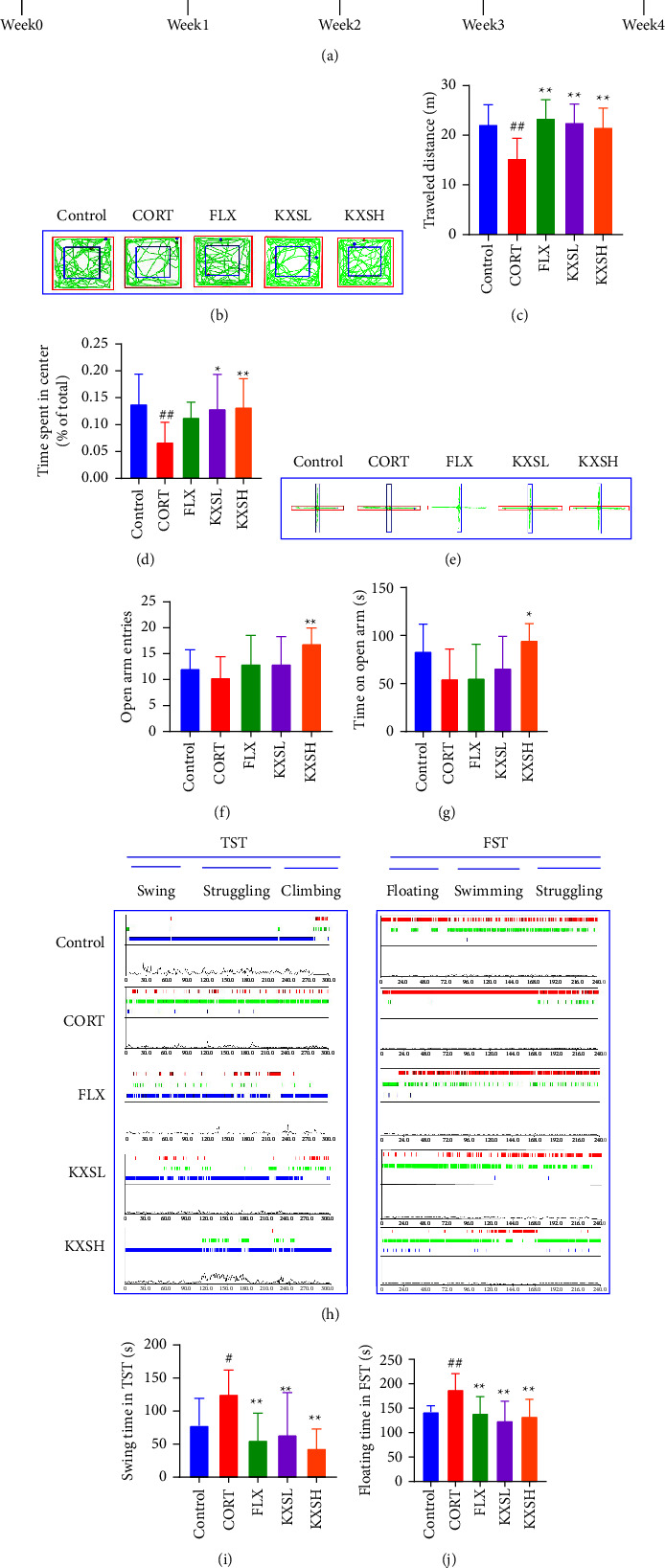
KXS ameliorated CORT-induceddepression-like behaviors. (a) The Design of the experiment. (b) Representative tracks in OFT. (c) and (d) Travel distance and time spent in center statistic results in OFT (*n* = 12). (e) Representative tracks in EPM. (f) and (g) Numbers into open arm and time on open arm statistic results in EPM (*n* = 12). (h) Representative tracks in TST and FST. (i) Swing immobility time in TST (*n* = 12). (j) Floating immobility time in FST (*n* = 12). The data were presented as the mean ± SEM. ^##^*P* < 0.01, ^#^*P* < 0.05 compared with the control group; ^*∗∗*^*P* < 0.01, ^*∗*^*P* < 0.05 compared with the CORT group.

The experiment in this study used CORT (TCI, Japan) dissolved in saline solution containing 0.1% dimethyl sulfoxide and 0.3% Tween 80. CORT, KXSL, KXSH, and FLX group mice received 20 mg/kg/d of CORT suspension through subcutaneous injection for three consecutive weeks to induce depression in the depression model, as described in previous studies [20]. FLX was selected as a positive control to validate the efficacy of KXS treatment, while mice in the CON group received saline that contained 0.1 percent dimethylsulfoxide and 0.3 percent Tween-80. From the week 2 to week 3, on the basis of CORT injection, the KXSL and KXSH groups were given KXS extracted solution 0.35 g/kg/d and 0.7 g/kg/d, respectively, by gavage, and the FLX group was intragastrically administered FLX (15 mg/kg/d, macklin, China). While an equal volume of saline was given in the CON group mice (Figure 1(a)).

### 2.4. Behavioral Tests

At the end of treatment, behavioral tests (open field, elevated plus maze, tail suspension, and forced swim test) were performed to assess the different aspects of depression.

#### 2.4.1. Open Field Test (OFT)

This experiment was done in an open box structure (50 × 50 × 40 cm) with a black square at the bottom. The mouse was placed in the center of the square and allowed to move freely for 6 min in the open field. Test time consisted of 1 minute of acclimation and 5 minutes of actual testing [21]. Video cameras positioned directly above the arena tracked the animal movements and recorded them on a computer with software (Super Maze, China) that determined the distance traveled and time spent in the center of the chamber compared to the edges. Mice acclimated to the room for 1 h before the test and the arena was cleaned with 70% EtOH after every test.

#### 2.4.2. Elevated plus Maze (EPM)

The elevated plus maze is a plus-shaped apparatus, which has a center quadrant of a four arms maze with a pair of open arms without walls and a pair of closed arms with walls (35 cm long, 6 cm wide). To track the movement of each mouse, a video camera was mounted directly over the arena, and video recordings were made on a computer with software (like OFT). The times of mice entering the open and close arms and the retention time in both arms were recorded by the software throughout a 5 min session. All measurements were conducted in a dimly lit experimental room in which the mice had been acclimatized for 1 hour prior to testing.

#### 2.4.3. Tail Suspension Test (TST)

TST experiment procedure was conducted to reference use of mice in accordance with Steru [22]. The whole test process took 6 minutes, and the mouse behavior was recorded with a high-definition camera. Motor tracking system (as with OFT) was used to analyze the videos, and the immobility period was calculated cumulatively during the last 5 min [23].

#### 2.4.4. Forced Swim Test (FST)

The trial was carried out in a transparent plexiglass cylinder (30 cm high ×18 cm diameter) filled with 10 cm depth of freshwater (23 ± 2°C) for 6 min. High-definition cameras were used to record the behavior of mice. And during the last 4 min, the immobility time was measured (define the first 2 min as habituation) by the motor tracking system (as above) of the test.

### 2.5. Brain Tissue Samples Preparation

After the completion of behavioral tests, mice (*n* = 6/group) were anesthetized and then transcardially perfused with PBS, flash frozen in liquid nitrogen, and stored at −80°C. Meanwhile, others mice were infused with PBS-buffered 4% paraformaldehyde (PFA) to extract brain for slice staining examination.

### 2.6. Nissl Staining

In the study, paraffin sections of the brain (5 *µ*m) were washed in xylene and rehydrated by graded ethanol and double-distilled water. Then, these sections were immersed in Nissl's stain at room temperature for 15 min. After rinsing with double-distilled water and dehydrating with 70%, 95%, and 100% gradient alcohol, the slides were removed in xylene and sealed with neutral gum. Lastly, neurons and Nissl bodies in the hippocampus CA3 area (HIPP) and medial Prefrontal cortex (mPFC) were observed by microscope (Nikon 80i, Japan).

### 2.7. Senescence *β*-Galactosidase Staining

The units of *β*-galactosidase activity were determined according to the manufacturer's instructions using Senescence's *β*-galactosidase Staining Kit (Beyotime Biotechnology, China). Frozen sections (30 *µ*m) were fixed for 15 minutes with the fixative at room temperature. Afterward, they were washed three times with 0.1 ml PBS (pH 7.4) and incubated overnight at 37°C in freshly prepared staining buffer. Images were then analyzed using a microscope (Nikon 80i, Japan).

### 2.8. Immunofluorescence Staining

Brain cryosections (30 *µ*m) were incubated in 0.3% Triton-X for 20 min and a PBS-buffered blocking solution containing 10% normal goat serum, 30 min, followed by incubation in a primary antibody solution containing anti-Iba1 antibody (1 : 400, rabbit, Reagent, #CAF6806), and were incubated at 4°C for 48 h. After three washes with PBS for 5 min each time, slices were incubated with fluorescence-labeled secondary antibodies, shielded from light and incubated at room temperature for 2 h. Then, after three washes with PBS for 5 min each time, DAPI (1.0 *μ*g/ml) was applied for 10 min. Images were obtained with an inverted fluorescence microscope (DMi8, Leica, Germany).

### 2.9. Transcriptome Sequencing and Data Analysis

RNA samples were isolated using the Ribo-Zero rRNA Removal Kit (Epicentre, Madison, WI, USA). Each sample, which was divided into 1.0 *μ*g RNA and 3.0 *μ*g DEPC water and stored in dry ice, was sent for RNA sequencing. The sequencing libraries were prepared using NEBNextR UltraTM Directional RNA Library Prep Kit from IlluminaR (NEB, USA), according to the manufacturer's instructions. Each sample's sequence was assigned an index code, and PCR products were purified with the AMPLEXP system. The Agilent Bioanalyzer 2100 was used to analyze the library quality, and qPCR was used to determine the quality of the PCR products. On the acBot Cluster Generation System, the TruSeq PE Cluster Kitv3-cBot-HS (Illumina) was used to cluster the index-coded samples following the manufacturer's instructions. Following cluster generation, library preparations were sequenced on an Illumina Hiseq platform to generate paired-end reads. The total RNA from each tissue was used to build libraries for transcriptome sequencing. The library construction and sequencing occurred at BGI‐Shenzhen, China.

### 2.10. Immunohistochemistry (IHC)

The SABC IHC staining kit (Boster, China) was used for IHC staining of 4-HNE following the manufacturer's protocol. IHC staining was performed after sequentially deparaffinizing and hydrating brain paraffin sections (5 *µ*m) with xylene, ethanol, and ethanol of graded concentrations. Rabbit polyclonal IgG anti-4-HNE (1 : 200, Bioss, #BA02267845) was used in this experiment at a dilution of 1 : 200. Staining with DAB and observation under a microscope (Nikon 80i, Japan) confirmed the presence of positive immunoreactivity. The IHC staining was scored according to the immunostaining intensity as previously described [24].

### 2.11. MDA and SOD Measurement

Ultrasonic waves were used to homogenize snap-frozen brain tissue. An aliquot of the homogenate was used to assay for MDA formation and SOD activity. Using the Lipid Peroxidation MDA Assay Kit (Beyotime, China) and the SOD Assay Kit (Nanjing Kaiji Bio-tech Co., China), manufacturers' instructions were followed for measuring MDA levels and SOD activity.

### 2.12. Detection of ROS and JC-1 Staining *in Vivo and in Vitro*

To evaluate the effect of KXS on ROS generation and mitochondrial membrane potential, the fluorescent probe dihydroethidium (DHE) kit (Beyotime, China), CellROX® Oxidative Stress kit (CellROX) and JC-1 Mitochondrial Membrane Potential Assay Kit (Abcam, ab113850) were used to examine the ROS level and alternations of mitochondrial membrane potential in CORT-induced mice or LPS/IFN*ɣ*-induced BV2 cells following the manufacturer's instructions [25, 26]. Images were obtained with an inverted fluorescence microscope (DMi8, Leica, Germany).

### 2.13. Cell Culture and Design

A cell culture incubator with 5% CO_2_ was used for cell culture of BV2 cells (Wuhan University Cell Bank, China) at 37°C in DMEM with 10% FBS and antibiotics (1% penicillin and streptomycin). To determine how KXS influenced the inflammatory response, BV2 cells were pretreated with or without corresponding concentrations of KXS (200 *μ*g/mL) for 2 h, then followed by exposure to LPS (100 ng/mL)/IFN*γ* (25 ng/mL) for another 24 h.

### 2.14. Assay of NO

NO release into the medium was measured using the NO assay kit (Beyotime, China) according to the instructions. Using an absorbance of 540 nm and a standard curve for correction, NO concentration was measured with a microplate reader (Multiskan FC, Thermoscience, United States).

### 2.15. Real‐Time Quantitative Fluorescence PCR (RT-qPCR)

The total RNA was extracted using RNAiso Plus (Takara, China), and RT‐qPCR analysis was performed using TB Green® Premix Ex TaqTM II (Takara, China) as directed. Quantification reactions were carried out in triplicate for each sample using a standard curve method. The amplification cycles were 95°C for 5 s, 60°C for 20 s, and 72°C for 15 s. Finally, a melting curve was constructed to evaluate the specificity of the reaction. In the current study, GAPDH and Actb were used as housekeeping genes and the relative mRNA concentrations were measured by *E* = 2^−ΔΔCt^. The following primers appear in Table 2.

### 2.16. Statistical Analyses

Data in this study are expressed as mean ± SEM. Differences among groups have been analyzed using one-way analysis of variance followed by Dunnett's test. Analyses of the data were conducted using Prism 8.0, and *P* values less than 0.05 were perceived to be statistically significant.

## 3. Results

### 3.1. KXS Ameliorated CORT-Induced Depressive-Like Behavior

OFT test displayed that KXSL and KXSH therapy markedly enhanced the travel distance (*P* < 0.01, *P* < 0.01) and the percentage of time spent in the center (*P* < 0.05, *P* < 0.01) in relation to the CORT group (Figures 1(b)–1(d)). Likewise, in comparison with the CORT group, KXSH treatment significantly improved the number of entries (*P* < 0.01) and time (*P* < 0.05) to the open arms of the EPM ((Figures 1(e)–1(g)). These results demonstrated that CORT impeded locomotor activity and spontaneous exploratory behavior, which were effectively reverted after KXS treatment. Then, the representative tracks of the TST and FST are shown in Figure 1(h). According to TST, CORT administration significantly increased mice's immobility time when compared with the control (*P* < 0.05); however, KXS and FLX administration could effectively reduce the immobility time (*P* < 0.01, *P* < 0.01, Figure 1(i)). Similarly, KXS and FLX administration significantly reduced the immobility time in FST compared with the CORT group (*P* < 0.01, *P* < 0.01, Figure 1(j)). All these results suggested that KXS treatment could be able to efficiently alleviate CORT-induced despair in mice.

### 3.2. KXS Improved CORT-Induced Neuronal Damage in Mice

**Figure 2:**
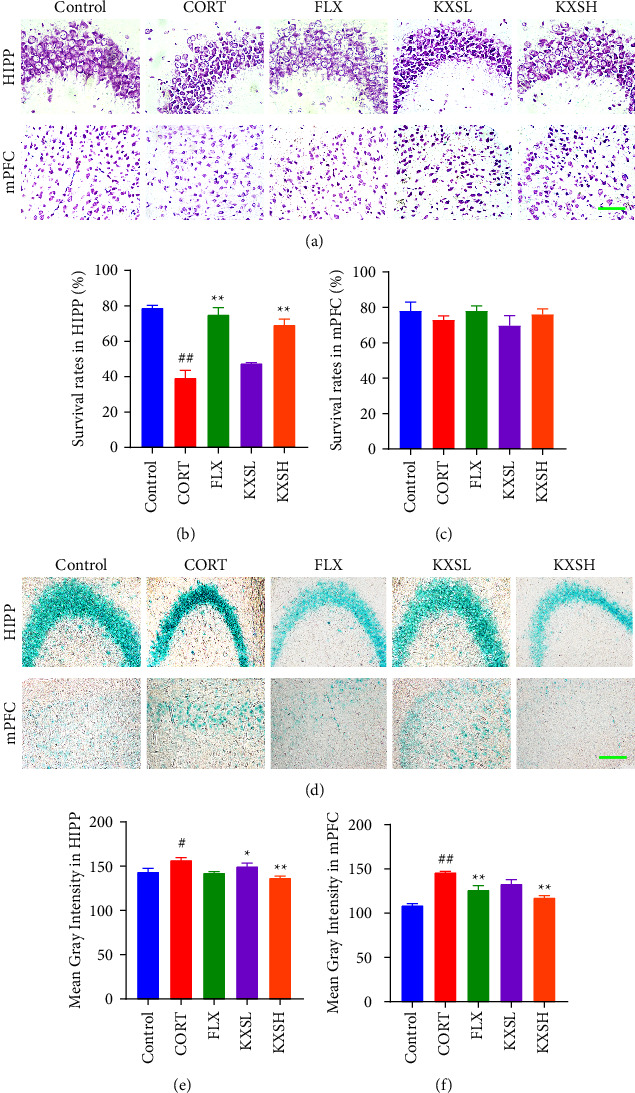
Effect of KXS on CORT-induced neuronal damage mice. (a) Nissl staining in HIPP and mPFC of the brain. Bar = 400 *μ*m. (b) and (c) Quantification of the numbers of Nissl-stained neurons by survival neuron rates in HIPP and mPFC of the brain (*n* = 4). (d) *β*-Galactosidase staining in HIPP and mPFC of the brain. Bar = 200 *μ*m. (e) and (f) Mean gray value statistics in HIPP and mPFC of *β*-galactosidase staining (*n* = 4). The data were presented as the mean ± SEM. ^##^*P* < 0.01, ^#^*P* < 0.05 compared with the control group; ^*∗∗*^*P* < 0.01, ^*∗*^*P* < 0.05 compared with the CORT group.

Nissl staining and *β*-galactosidase staining were used to investigate whether CORT damaged the neurons. Compared to the control group, CORT group clearly showed neuronal damage in HIPP (*P* < 0.01). FLX and KXSH obviously ameliorated the survival neuron rates of HIPP neurons of mice compared with CORT group (*P* < 0.01, *P* < 0.01, Figures 2(a)–2(c)). The same result was observed in *β*-galactosidase staining (Figures 2(d)–2(f)). Compared with the control, CORT group obviously showed neuronal senescence in HIPP and mPFC (*P* < 0.05, *P* < 0.01). KXSH treatment significantly ameliorated the neuronal senescence in HIPP and mPFC (*P* < 0.01, *P* < 0.01). All of these results indicated that KXS could alleviate CORT-induced neuronal damage of depression mice.

### 3.3. Transcriptomics Data Analysis Showed That KXS Regulated CORT-Induced Depression in Mice Mainly through the Immune System and Signal Transduction Pathways

**Figure 3:**
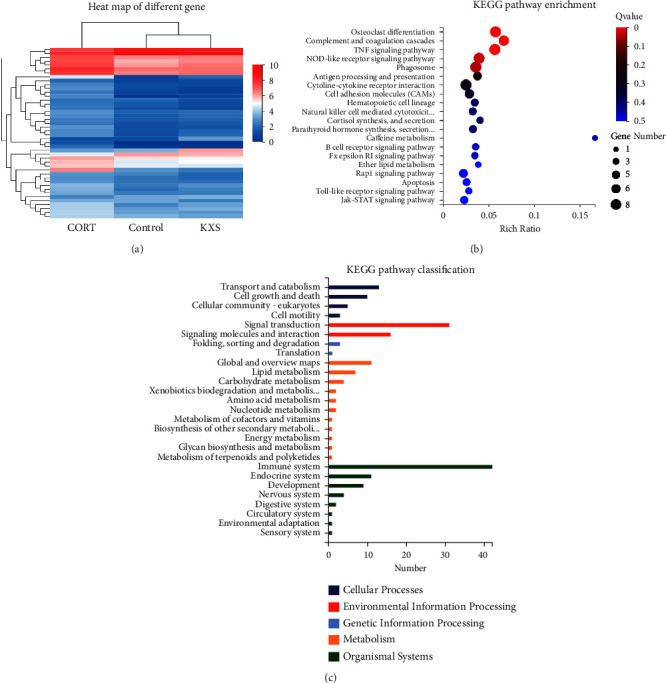
Effect of KXS transcriptomics data analysis in heat map and KEGG. (a) Heat map of differential gene expression analysis in Control, CORT, and KXS groups. (|log2FC| ≥1, difference ≥2-fold; blue and red colors representing downregulated and upregulated expression, respectively). (b) and (c) KEGG pathway enrichment and classification analysis shows that KXS majorly regulated the genes involved in through immune system and signal transduction pathways (|log2FC| ≥1, *Q* value ≤0.05).

**Figure 4:**
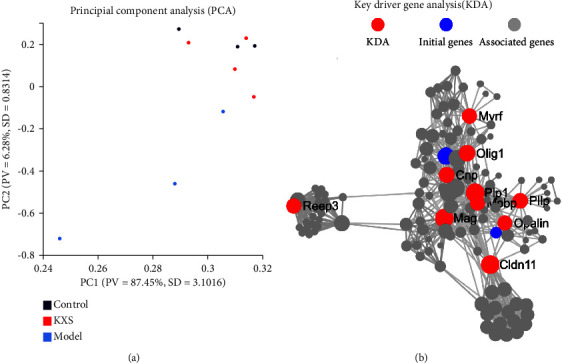
Effect of KXS transcriptomics data analysis on CORT-induced depression in PCA and KDA. (a) Results of Principal component analysis (PCA) in Control, CORT, and KXS groups. (The dots represent the samples, and the same color represents the same sample group. PV represents Proportion of variance and SD represents standard deviation). (b) Key driver gene analysis (KDA) of PPI network. Red node represents key driver genes, blue node represents initial genes, and gray node represents related genes with them.

To further understand the therapeutic mechanism of KXS in the treatment of depression, transcriptome analysis was performed on the Control, CORT, and KXS group. Heat map of differential gene expression analysis identified that there were 44 differentially expressed genes between CORT and KXS groups. Among them, six genes were upregulated, while 38 genes were downregulated. Apparently, it can be seen that the KXS group significantly reversed the differential gene expression between the CORT group and the control group (Figure 3(a)). By analyzing differentially expressed genes, GO functional enrichment analysis of differential genes was used for attribution of functions to KXS regulation. KEGG analysis indicated that KXS majorly regulated the genes involved in through signal immune system (Osteoclast differentiation, complement cascades, TNF, etc.) and transduction pathways (NOD-like receptor, Antigen processing, etc.) (Figures 3(b)–3(c)). To further explore the depression regulatory mechanisms, we focused on the principal component analysis (PCA) and KDA of differential genes. The results of PCA displayed that there were 19 Principal components, and KXS significantly reversed the differential PCA expression between the CORT group and the control group (Figure 4(a) and Table 3). The KDA showed that there were 10 genes in the main regulatory position (Figure 4(b) and Table 4). Surprisingly, the key gene Pllp is both the principal component and the key target, which suggests that it may be the key target of KXS in the treatment of CORT-induced depression. All these data postulated that KXS attenuated CORT-induced depression in mice mainly through regulating immune, inflammatory, and signal transduction pathways.

### 3.4. KXS Inhibited CORT-Induced Microglia Activation and Inflammatory Response

**Figure 5:**
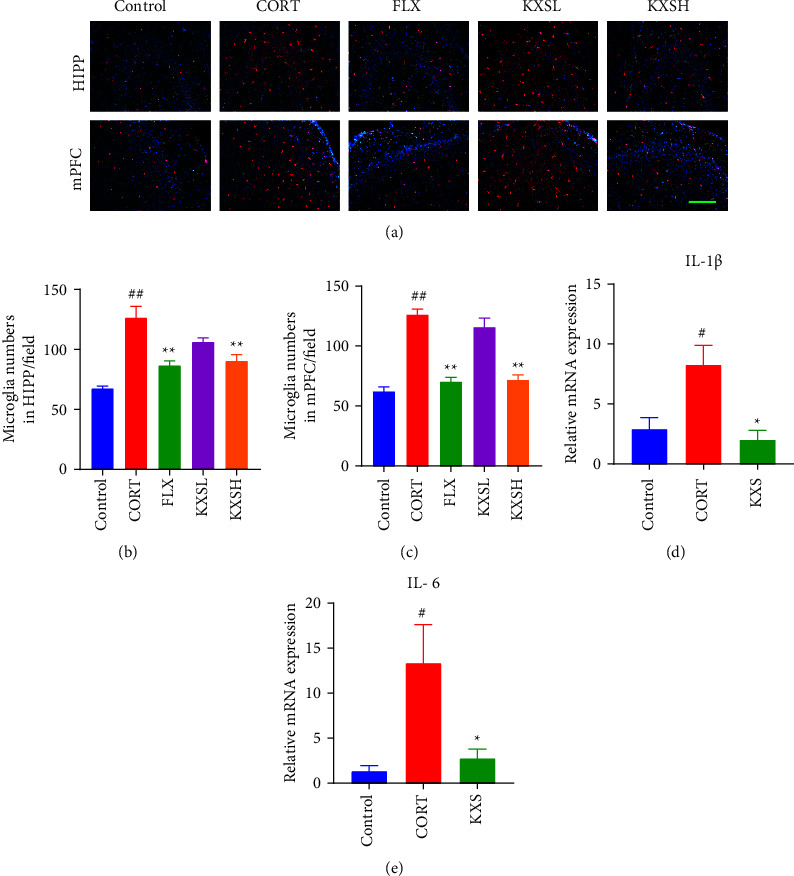
Effect of KXS on CORT-induced microglia activation and inflammatory activation response. (a) The fluorescence micrographs of microglia of the brain in HIPP and mPFC with Iba1 antibodies (red) and DAPI (blue) for nuclei. Bar = 200 *μ*m. (b) and (c) Quantification of the number of Iba1^+^ cells in HIPP and mPFC (*n* = 4). (d) and (e) Relative inflammatory factors expression statistics of the IL-1*β* and IL-6 by RT-qPCR in mouse brain tissue (*n* = 3). The data were presented as the mean ± SEM. ^##^*P* < 0.01, ^#^*P* < 0.05 compared with the control group; ^*∗∗*^*P* < 0.01, ^*∗*^*P* < 0.05 compared with the CORT group.

Iba1 immunofluorescence staining was performed to detect microglial activation; and the inflammatory factors expression of IL-1*β* and IL-6 were measured by RT-qPCR in mouse brain tissue. Compared with the control group, CORT group obviously increased the number of microglia in HIPP and mPFC (*P* < 0.01, *P* < 0.01). In contrast to the CORT group, FLX and KXSH treatment significantly decreased the number of microglia in HIPP and mPFC (*P* < 0.01, *P* < 0.01, Figures 5(a)–5(c)). Similar data were shown in the inflammatory factor expression of RT-qPCR. The administration of KXS clearly decreased the inflammatory factors overexpression of IL-1*β* and IL-6 in HIPP of mice compared to CORT group (*P* < 0.05, *P* < 0.05, Figures 5(d)–5(e)). All of these data indicated that KXS ameliorated CORT-induced microglia activation and decreased the inflammatory response in depression mice.

### 3.5. KXS Defended Against CORT-Induced Oxidative Stress Response of Mouse Brain Tissue

**Figure 6:**
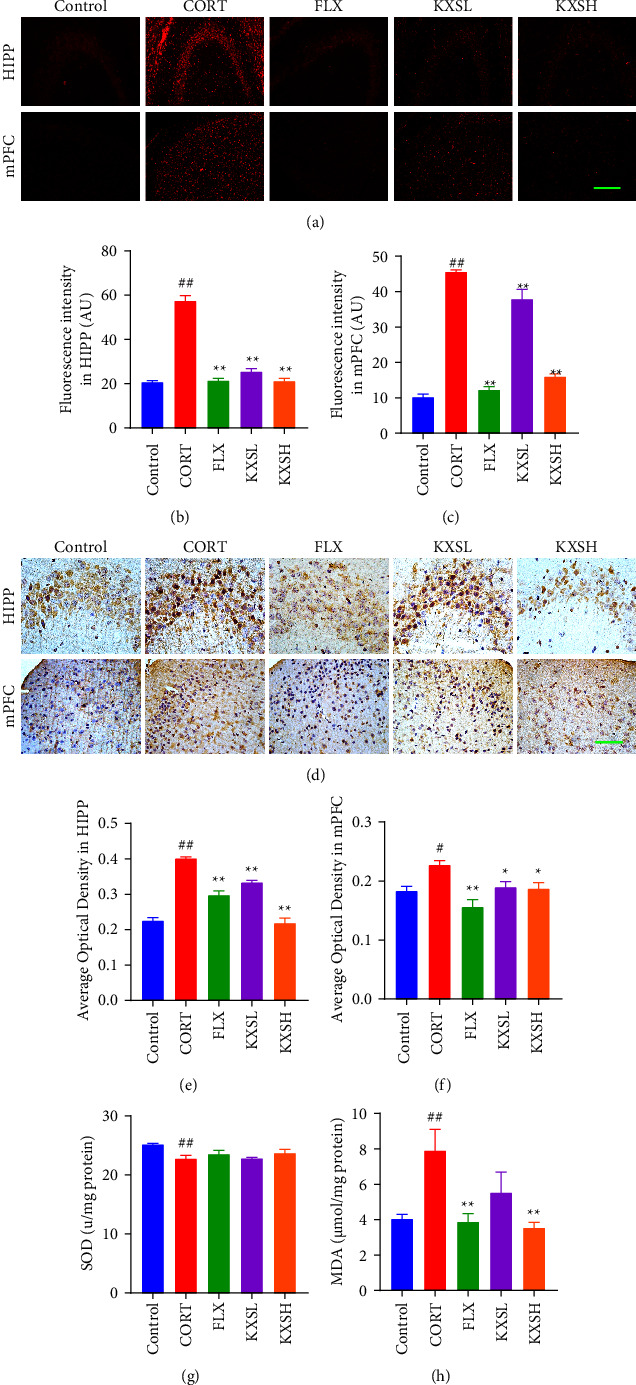
Effect of KXS in CORT-induced oxidative stress response of mouse brain tissue. (a) DHE staining in HIPP and mPFC of the brain. Bar = 200 *μ*m. (b) and (c) The fluorescence intensity of DHE represents the ROS concentration in the brain's HIPP and mPFC. (*n* = 4). (d) IHC staining of 4-HNE in HIPP and mPFC of the brain. Bar = 400 *μ*m. (e) and (f) Average optical density of 4HNE in HIPP and mPFC of the brain (*n* = 4). (g) Total SOD activity was determined by a Kit-WST. (*n* = 5). (h) The content of MDA in brain was determined by lipid peroxidation index (*n* = 5). The data were presented as the mean ± SEM. ^##^*P* < 0.01, ^#^*P* < 0.05 compared with the control group; ^*∗∗*^*P* < 0.01, ^*∗*^*P* < 0.05 compared with the CORT group.

To demonstrate whether the anti-oxidative property was included in the neuroprotective effect of KXS, we performed the DHE staining, IHC staining of 4-HNE, oxidative stress product MDA content, and activity of antioxidant SOD to detect ROS concentration. DHE staining showed that CORT administration significantly increased the ROS concentration (*P* < 0.01, *P* < 0.01) of mouse brain tissue compared with the control group in HIPP and mPFC, while FLX and KXS treatment effectively reversed this effect (*P* < 0.01, *P* < 0.01, Figures 6(a)–6(c)). Moreover, compared to the control, CORT administration obviously increased the expression of 4-HNE (*P* < 0.01, *P* < 0.05, Figures 6(d)–6(f)). By oxidizing lipids in the cell, lipid peroxidation is considered an important indicator of oxidative stress, in HIPP and mPFC. Compared to the CORT group, KXS remarkably decreased the expression of 4-HNE (*P* < 0.01, *P* < 0.05, Figures 6(d)–6(f)) in HIPP and mPFC in the mouse brain. Furthermore, compared with the control, CORT administration obviously decreased the expression of SOD (*P* < 0.01, Figure 6(g)), and increased the expression of MDA (*P* < 0.01, Figure 6(h)). Compared to the CORT group, KXSH remarkably decreased the expression of MDA (*P* < 0.01, Figure 6(h)) in the mouse brain. These results indicated that KXS effectively defended against CORT-induced oxidative stress and thereby acted as neuroprotective effects on mouse brain tissue.

### 3.6. KXS Suppressed LPS/Ifn*γ*-Induced Inflammatory Activation of BV2 Microglia Cells

**Figure 7:**
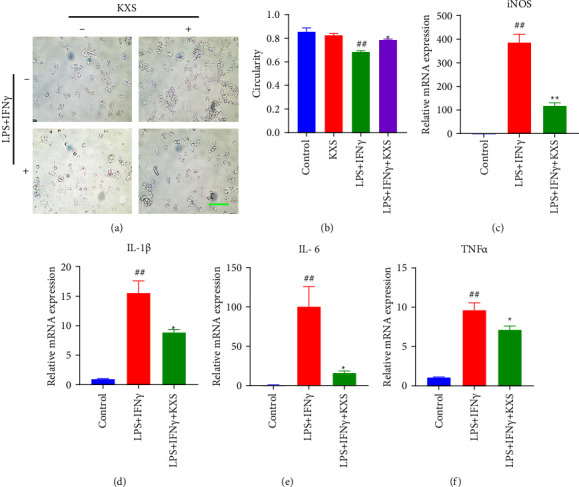
Effect of KXS on LPS/IFN*ɣ*-induced inflammatory activation response in BV2 cells. (a) LPS/IFN*γ*-induced inflammatory activated morphology in BV2 cells. Bar = 200 *μ*m. (b) The average circularity of BV2 cells (*n* = 3). (c)–(f) Relative BV2 cells inflammatory factors expression statistics of the iNOS, IL-1*β*, IL-6, and TNF*α* by RT-qPCR (*n* = 3). The data were presented as the mean ± SEM. ^##^*P* < 0.01 compared with the control group; ^*∗∗*^*P* < 0.01, ^*∗*^*P* < 0.05 compared with the LPS/IFN*γ* group.

BV2 cell morphology results showed that in the control group, microglia cells were small and round with regular shape; in the LPS + IFN*γ* group, microglia cells were adhered, swollen, and their volume increased with irregular shape, which formed a slender pseudopod; KXS 200 *μ*g/mL administration reduced microglial adhesion and pseudopodia, decreased cell volume, and increased cell roundness (Figure 7(a)). Likewise, KXS significantly reversed LPS/IFN*γ*-induced decrease of cell circularity in BV2 cells (*P* < 0.05, Figure 7(b)). Next, the mRNA expression levels of iNOS, IL-1*β*, IL-16, and TNF*α* were measured using RT-qPCR to detect the effect of KXS on the inflammatory activation response (Figures 7(c)–7(f)). Compared to the control, CORT treatment obviously increased (*P* < 0.01) the mRNA expression of iNOS, IL-1*β*, IL-16, and TNF*α*. In contrast to the CORT group, KXS significantly inhibited the mRNA expression levels of LPS/IFN*γ*-induced IL-1*β* (*P* < 0.05), IL-16 (*P* < 0.05), TNF*α* (*P* < 0.05), and iNOS (*P* < 0.01). These data indicated that KXS significantly decreased LPS/IFN*γ*-induced microglia (M1) inflammatory activation response [27].

### 3.7. KXS Prevented LPS/Ifn*γ*-Induced Oxidative Stress and Mitochondrial Dysfunction in BV2 Microglia Cells

**Figure 8:**
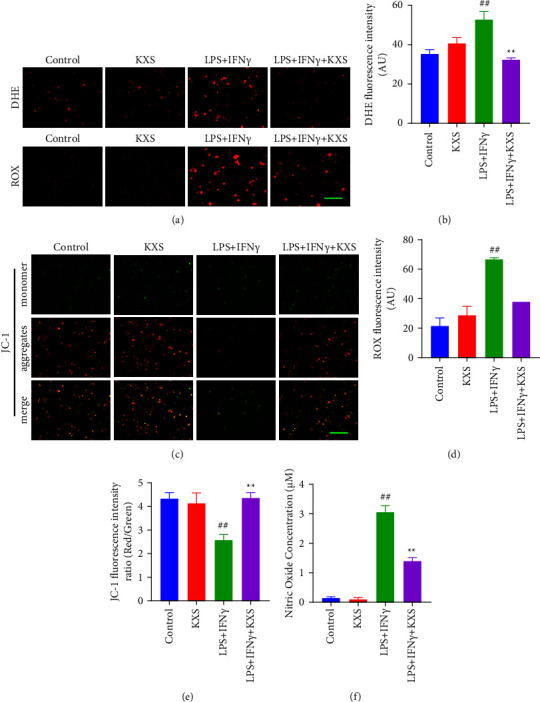
Effect of KXS in LPS/IFN*γ*-induced oxidative stress and mitochondrial dysfunction of BV2 cells. (a) BV2 cells are analyzed for ROS concentration using DHE and ROX fluorescence. Bar = 200 *μ*m. (b) and (c) Relative fluorescence intensity statistics of the content of DHE and ROX in BV2 cells (*n* = 3). (d) The mitochondrial membrane potential was detected by JC-1 staining. Bar = 200 *μ*m. (e) An analysis of relative fluorescence intensity of the red to green expression ratio (Red/Green) in BV2 cells (*n* = 4). (f) The effect of KXS on the NO concentration of BV2 cells was examined by NO assay (*n* = 3). The data were presented as the mean ± SEM. ^##^*P* < 0.01 compared with the control group; ^*∗∗*^*P* < 0.01 compared with the LPS/IFN*γ* group.

The mitochondrial membrane potential as well as ROS generation by DHE staining, ROX staining, and JC-1 staining were examined to demonstrate whether KXS could protect LPS/IFN*γ*-induced BV2 cells from mitochondria damage by anti-oxidative properties. The excessive ROS production was revealed by DHE staining and ROX staining in LPS/IFN*γ*-induced BV2 cells (*P* < 0.01, *P* < 0.01), and the administration of KXS efficiently reversed the excessive ROS production (*P* < 0.01, *P* < 0.01, Figures 8(a)–8(c)). Similar results were observed in JC-1 staining of LPS/IFN*γ*-induced BV2 cells (Figures 8(d)–8(e)). Compared with the control, LPS/IFN*γ* treatment obviously decreased the aggregates expression and increased monomer expression (the expression ratio, *P* < 0.01). In contrast to the LPS/IFN*γ* group, KXS significantly increased the aggregates expression and decreased monomer expression (the expression ratio, *P* < 0.01). NO assay results also showed that LPS/IFN*γ* increased cell NO concentration (*P* < 0.01), while KXS decreased NO concentration (*P* < 0.01, Figure 8(f)). All of these data suggested that KXS effectively suppressed the LPS/IFN*γ*-induced oxidative stress and decreased the damage of mitochondrial membrane potential in BV2 cells.

## 4. Discussion

In the current research, we verified the efficacy of KXS treatment by evaluating depressive-like behavior and neuronal damage in a CORT-induced depressive mouse model. According to the Transcriptome Data Analysis, we found that KXS, mainly by regulating the immune system (Osteoclast differentiation, complement cascades, TNF, etc.) and transduction pathways (NOD-like receptor, Antigen processing, etc.) reduced CORT-induced depression in mice; and there were 10 KDA genes (Cnp, Pllp, etc.), especially Pllp (mainly related to the immune mechanism of apical endocytosis controls [28]), with the strongest correlation in regulating depression of mice. Based on these transcriptome data results, we performed *in vivo* and *in vitro* experiments to verify the results. KXS effectively inhibited CORT-induced inflammatory activation and oxidative stress in mouse brain tissue. Further experiments revealed that KXS significantly decreased the mRNA content levels of LPS/IFN*γ*-induced IL-1*β*, IL-16, TNF*α* and iNOS, and protected LPS/IFN*γ*-Induced from mitochondria damage by anti-oxidative properties. All these data strongly indicated that KXS could decrease CORT-induced neuronal damage in depression mice by inhibiting neuroinflammation and oxidative stress.

Previous studies pointed that the increase of CORT levels resulted in the damage of hippocampal neurons and induced the depressive-like behavior of mice [29–31]. KXS significantly improved the travel distance of mice in OFT and decreased the immobility time in FST and TST [10, 32]. In this research, we successfully induced depressive-like behavior of mice by CORT administration, and showed neuronal damage in HIPP of CORT mice, while these effects were reversed by KXS. These results demonstrated that KXS presented antidepressant effects by ameliorating CORT-induceddepressive-like behavior and neuronal degenerative damage in mice.

Activated microglia are able to produce inflammatory mediators and reactive oxygen species which can lead to tissue injury and neurotoxicity through mechanisms such as oxidative stress [33]. The excessive secretion of corticotropin-releasing factor induces the dysfunction of HPA axis and leads to the damaged cortisol negative feedback mechanism [34]. Microglia can be activated and instigate an inflammatory response in the central nervous system when cortisol levels are high [35, 36]. Once activated, microglia will be contributed to pathology by promoting neuroinflammation; as a result, microglial activation induces the development of depression [37]. Microglia express pattern recognition receptors (PRRs) and thus identify the pathogen and damage-related molecular pattern molecules. In response to ligand binding to PRRs, they acquire amoeboid-like features, migrate to sites of inflammation, and release proinflammatory factors and neurotoxic factors such as NO produced by inducible NO synthase (iNOS) and ROS [38–41]. A key mechanism of KXS antidepressant effects is its modulation of the gut-brain axis, which includes gut microenvironment modification, suppression of neuronal inflammation, and inhibition of the HPA axis [42]. Luo et al. reported that the effects of KXS on cognitive dysfunction have been attributed to inhibiting microglial activation and inflammation levels of IL-6, IL-1*β*, and TNF*α* in the brain's cortex and hippocampus [43]. The elevated levels of MDA, the marker of oxidative damage to fatty acids, were detected in depressive patients [44, 45]. Ginsenoside-Rg1 attenuated oxidative stress by reducing the expression of 4-HNE, MDA content, ROS, and NO production, while improving the SOD activity in the hippocampal of depressed rats [46]. Consistent with this, our results also showed that KXS significantly reversed CORT-induced microglia activation and proinflammatory response of IL-1*β*, IL-6, and TNF*α* in mouse brain tissue and moreover, KXS effectively decreased the CORT-induced senescence, the expression of 4-HNE, MDA content, and the production of ROS, while increasing the SOD activity in mouse brain tissue. These results indicated that KXS efficiently inhibited CORT-induced microglia inflammatory activation and oxidative stress, thereby acting as neuroprotective effects on mouse brain tissue.

Studies have demonstrated an association between depression and increased oxidative stress and inflammation and, consequently, an increase in neuronal apoptosis [47, 48]. Owing to the function of producing energy in the electron transport chain (ETC), mitochondria are the main source of ROS [49, 50]. The increased production of mitochondrial reactive oxygen species was also found in depression, which could prove the dysfunction of mitochondria [51, 52]. Rezin et al. demonstrated that complex I, III, and IV of ETC as well as creatine kinase were inhibited in depression rats' cerebral cortex and cerebellum [53]. ROS products produced by excessive oxidative stress can cause mitochondrial and DNA damage, thus triggering cell death in neurons and glia [46]. Increasing oxidative and nitrosative stress results in the accumulation of toxic molecules, including oxidizing lipids, proteins, and damaging nucleic acids which further activate the inflammatory response of neurons [54]. Our study displayed the LPS/IFN*γ*-induced microglia (M1) inflammatory activation response, the mitochondrial membrane potential loss by JC-1 staining; and the excessive ROS and NO production revealed with ROX staining and NO assay in BV2 cells. KXS prevented the upregulation of microglia inflammatory activation, oxidative stress, receded neuronal senescence, and damage. In addition, we found that the mitochondrial membrane potential impairments, which were increased by LPS/IFN*γ* exposure, were decreased by KXS treatment. According to these findings, KXS could provide antidepressant effects through its effect on oxidative stress, which possibly through mediation by ameliorating the mitochondrial membrane potential damage.

## 5. Conclusion

Taken together, our study presents strong evidence supporting that KXS improved the CORT-induceddepressive-like behaviors of mice by its neuroprotective effects in inhibiting microglia activation and reduced the damage of mitochondria by anti-oxidative stress response. The suppression of the oxidative stress pathway appears to be responsible for at least part of these effects. These results uncovered the potential mechanisms for the neuroprotective effects of KXS against neuronal damage and provide insight into the possible development of novel therapeutic approaches for depression.

In our research, the KXS in the treatment of depression was only discussed from the phenotype of anti-inflammatory and antioxidant stress. However, the underlying mechanism of its key targets have not been studied in depth. Therefore, further exploration is needed on the basis of existing experimental results in the future study.

## Figures and Tables

**Table 1:** The herbal materials used for the preparation of KXS extracts.

Botanical name	Herbal name	Chinese name	Lot no.	Voucher no.
*Panax ginseng C. A. Meyer*	Ginseng	Ren shen	2102018	ZH20210203
*Poria cocos (Schw.) Wolf.*	Hoelen	Fu ling	2106078	ZH20210617
*Polygala tenuifolia Willd.*	Polygala	Yuan zhi	2103006	ZH20210302
*Acorus tatarinowii Schott*	Acorus	Shi changpu	D2101052	ZH20210120

**Table 2:** PCR primer sequences.

mRNA	Forward primer (5′-3′)	Reverse primer (5′-3′)
IL-1*β*	CTGTGACTCATGGGATGATGATG	CGGAGCCTGTAGTGCAGTTG
IL-6	CTGCAAGAGACTTCCATCCAG	AGTGGTATAGACAGGTCTGTTGG
TNF*α*	CAGGCGGTGCCTATGTCTC	CGATCACCCCGAAGTTCAGTAG
iNOS	ACATCGACCCGTCCACAGTAT	CAGAGGGGTAGGCTTGTCTC
GAPDH	AGGTCGGTGTGAACGGATTTG	GGGGTCGTTGATGGCAACA
Actb	GTGACGTTGACATCCGTAAAGA	GCCGGACTCATCGTACTCC

**Table 3:** Results of Principal component analysis (PCA).

Gene symbol	Gene ID	Qvalue (model/Control)	Qvalue (KXS/Model)
105244980	Gm40498	2.53*E − *04	8.90*E − *02
105244999	Gm40514	6.09*E − *06	1.92*E − *02
106648	Cyp4f15	1.73*E − *02	1.39*E − *02
12628	Cfh	6.37*E − *01	6.48*E − *01
12870	Cp	6.30*E − *01	3.65*E − *01
13653	Egr1	3.94*E − *18	4.86*E − *02
15360	Hmgcs2	3.06*E − *02	8.20*E − *03
18414	Osmr	6.49*E − *03	4.29*E − *02
18976	Pomc	2.65*E − *02	2.38*E − *01
20311	Cxcl5	3.11*E − *01	6.31*E − *01
226777	C130074G19Rik	8.35*E − *03	1.10*E − *01
230379	Acer2	1.93*E − *02	1.64*E − *04
239766	Rtp1	4.11*E − *	3.49*E − *02
242864	Napepld	3.66*E − *03	3.49*E − *02
243911	Kirrel2	7.76*E − *01	1.86*E − *02
319734	Cacna2d4	3.01*E − *01	1.97*E − *01
320981	Enpp6	5.72*E − *03	1.64*E − *04
67801	Pllp	1.20*E − *03	1.82*E − *02
71760	Etnppl	3.74*E − *04	1.13*E − *03

**Table 4:** Results of key driver gene analysis (KDA).

Genesymbol	Gene ID	Qvalue (model/Control)	Qvalue (KXS/Model)
Cnp	12799	9.39*E − *04	3.28*E − *04
Mag	17136	2.13*E − *06	2.09*E − *03
Mobp	17433	9.08*E − *01	9.96*E − *01
Cldn11	18417	7.31*E − *07	4.92*E − *03
Plp1	18823	5.82*E − *04	2.09*E − *03
Myrf	225908	2.74*E − *07	1.03*E − *01
Opalin	226115	4.30*E − *01	3.99*E − *01
Reep3	28193	5.25*E − *01	9.96*E − *01
Olig1	50914	5.16*E − *04	1.86*E − *02
Pllp	67801	1.20*E − *03	1.82*E − *02

## Data Availability

The analyzed data used to support the findings of this study are available from the corresponding author upon request.
